# Accurate Botanical Nomenclature: Pomegranate and the ‘Aril’ Misconception

**DOI:** 10.3390/foods13020201

**Published:** 2024-01-08

**Authors:** Pablo Melgarejo, Juan José Martínez-Nicolás, Dámaris Núñez-Gómez, María Soledad Almansa, Pilar Legua

**Affiliations:** 1 Plant Production and Microbiology Department, Miguel Hernandez University, Ctra. Beniel 3.2 Km, 03312 Orihuela, Alicante, Spain; pablo.melgarejo@umh.es (P.M.); juanjose.martinez@umh.es (J.J.M.-N.); p.legua@umh.es (P.L.); 2 Plant Biology Department, Miguel Hernandez University, Ctra. Beniel 3.2 Km, 03312 Orihuela, Alicante, Spain; ms.almansa@umh.es

**Keywords:** *Punica granatum* L., exariled, botanical definitions, external testa, pomegranate seed, pomegranate aril

## Abstract

The pomegranate (*Punica granatum* L.) attracts attention in studies for its nutritional and medicinal properties. However, a recurring issue in the literature arises due to the multidisciplinary nature of these studies, leading to a mistaken repetition of basic botanical terms. The problem stems from the misapplication of the term “aril” to the pomegranate seed, despite the fruit being exariled, signifying the absence of an aril. This confusion may be attributed to the sarcotesta’s appearance, resembling a complete aril, coupled with a lack of awareness in fields such as medicine, pharmacy, and cosmetics. This study specifically examines the Kingdom-variety pomegranate, due its economic importance in the fruit market. The fruits were evaluated at different developmental stages—initial, intermediate, and commercial. Magnification photography techniques were used to study the development state of the pomegranate fruits. The physiological studies confirm that the pomegranate seed constitutes the complete grain, with the juicy, sweet part surrounding it identified as the testa, not an aril. The findings underscore a persistent error in the existing literature, emphasizing the necessity for dissemination and education in future studies. A thorough grasp of pomegranate anatomy and precise use of terminology are indispensable for ensuring accuracy and rigor in scientific communication.

## 1. Introduction

The pomegranate (*Punica granatum* L.) is an ancient cultivated plant, with its geographical roots traced to regions including Asia Minor, Transcaucasia, Iran, and Turkmenistan [1]. This fruit, native to the Middle East, has been valued throughout history for its nutritional and medicinal properties. Such attributes have captured the interest of the scientific community, leading to exploration of its benefits in various fields [2].

The pomegranate fruit holds significant economic value in the agricultural sector due to its medicinal and therapeutic properties. Currently, it is cultivated on approximately 300,000 hectares in countries like India, Iran, China, Turkey, and the United States [3]. Its adaptability to various climatic conditions and soil types has facilitated global dissemination. However, optimal production occurs in environments with high temperatures during the fruit ripening period (August–October).

In Spain, the pomegranate has gained significant prominence, representing the primary producer in Europe. As of 2022, the cultivated area reached approximately 5327 hectares, yielding 79,183 tons [4]. This production is predominantly concentrated in southern regions, responsible for 72% of the total.

Consumers highly esteem the pomegranate due to its unique organoleptic characteristics. The quality of this fruit is a delicate balance of taste attributes like sugars and organic acids along with nutraceutical compounds such as polyphenols [5,6]. These compounds, concentrated in the pulpy membrane of the seeds (sarcotesta) [7] can be consumed fresh or squeezed for juice.

Organic acids and sugars significantly influence the final sensory characteristics, impacting consumer preferences for sweet, sweet–tart, or tart genotypes. Additionally, the pomegranate contains a diverse range of polyphenolic compounds, including hydrolysable tannins, flavonoids, and phenolic acids [8]. These compounds have been associated with the prevention and treatment of various diseases, such as cancer, cardiovascular diseases, neurodegenerative diseases, and diabetes [8,9,10].

Beyond polyphenols, the pomegranate is considered healthy due to the presence of other phytochemical compounds like lipids, fiber, and minerals [6]. Previous research indicates an oil content ranging between 12% and 20% of the total seed weight, with unsaturated fatty acids predominating [3]. The raw seed fiber, reflecting the woody material compared to the total seed weight, provides an indication of overall seed palatability. Furthermore, studies highlight the abundance of minerals in pomegranate seeds, especially potassium, phosphorus, calcium, magnesium, and sodium [11,12]. Recent research also suggests that regular consumption of pomegranate may contribute to improving cardiovascular health, cognitive function, and reducing blood pressure [9,10].

Another intriguing aspect lies in the exploration of pomegranate’s potential in the cosmetics and personal care industry. The presence of polyphenolic compounds has sparked interest for topical application, observed to protect the skin against aging effects like wrinkles and spots [9,10,13]. Furthermore, antimicrobial properties have been explored, with certain pomegranate extracts showing effectiveness against bacteria and fungi [9,10,11,14].

The versatility and properties of pomegranate, largely due to its components, are corroborated by the versatility and multidisciplinarity of existing studies. However, despite the technical quality and scientific rigor of these articles, synthesizing the accumulated knowledge about this fruit reveals that many works contain errors and misinterpretations regarding basic botanical concepts associated with the pomegranate fruit.

### 1.1. Explanation of the Issue

In 2021, Melgarejo et al. [7] identified both the improper use of the term “aril” when referring to the entire pomegranate seed and the exclusive reference to the inner part of the seed as “seed” in the scientific literature. In the latter case, they observed that “aril” was exclusively used to describe the juicy pulp or outer covering of the seed. These errors were prevalent in the scientific community, indicating a fundamental misunderstanding of basic botanical concepts related to the pomegranate fruit.

**Figure 1:**
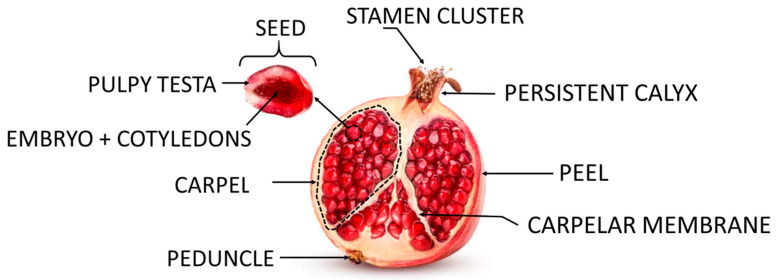
Pomegranate fruit parts.

The confusion in using the term “aril” to refer to the seed of the pomegranate may arise from the appearance of the sarcotesta—a thin layer with a juicy texture and a sweet and acidic taste. However, it is crucial to clarify that the pomegranate does not have arils as it is exarilate [7]. Instead, the juicy and sweet pulp of the pomegranate directly surrounds the central part, where the embryo with its cotyledons is located. This is the testa, or the external covering of the seed; as a whole, it constitutes the edible part of this fruit (Figure 1).

From a botanical standpoint, the aril is defined as a fleshy covering of certain seeds formed from the expansion of the funicle or the hilum. In contrast, the testa constitutes the outermost layer of the seed coat or integument surrounding the seed in seed plants, with the innermost layer referred to as the tegmen. Importantly, the testa originates from one of the ovule’s integuments known as the primina [15].

Despite the call for rectification in terminology in the 2021 publication [7], there has been no substantial progress or correction regarding the persistent terminological error surrounding pomegranate seeds. The use of the term “pomegranate aril” continues unabated. This enduring confusion is perpetuated through citations and is largely attributed to the diverse backgrounds of authors—coming from disciplines as varied as medicine, pharmacy, and food technology, where botanical knowledge may be limited or absent.

It is noteworthy that, at this point, direct citation of the works containing incorrect terminology is avoided to prevent potential discomfort or harm to the responsible authors. It is essential to acknowledge the scientific quality and rigor of these works and their contributions at all times. Our critique is solely centered on the “widespread” confusion regarding the appropriate terms for describing pomegranate seeds and their components.

### 1.2. Implications of This Terminological Error

The identified terminological error, using “aril” to refer to the pomegranate seed, holds significant implications for scientific research and communication:

- Confusion in Research Findings: Studies relying on the inaccurate terminology might have mischaracterized the parts of the pomegranate seed. This could lead to confusion in understanding the distribution of nutrients and bioactive compounds within the fruit.
- Inconsistency in the Literature: The persistent use of incorrect terminology might contribute to inconsistencies across the scientific literature. This could make it challenging for researchers to compare and integrate findings from different studies, hindering the advancement of knowledge in the field.
- Implications for Nutritional Studies: Nutritional studies that focus on specific components of the pomegranate, such as polyphenols in the sarcotesta, may be impacted by the misidentification of seed parts. Incorrectly attributing certain properties to the aril instead of the correct seed components could lead to inaccurate nutritional assessments.
- Misinterpretation in Medical and Health Research: Studies exploring the health benefits of pomegranate may be affected if the terminology error has led to a misunderstanding of which parts of the seed contain bioactive compounds with potential health benefits. This could influence recommendations for consumption and health interventions.
- Communication Challenges: The use of inconsistent and incorrect terminology creates challenges in effective communication among researchers, educators, and practitioners. Clear and accurate communication is essential for the dissemination of knowledge and the development of evidence-based practices.
- Difficulty in Building on Previous Work: Researchers building on previous studies may face challenges if the foundational knowledge is based on inaccurate terminology. This could slow down the progress of research in areas related to pomegranate properties and applications.
- Cross-Disciplinary Confusion: Given the diverse backgrounds of authors in disciplines such as medicine, pharmacy, and food technology, the confusion in botanical terminology may persist across different fields. This interdisciplinary confusion can hinder collaboration and the integration of knowledge from various domains.

The fundamental innovation of this article focuses on a comprehensive and pioneering approach to the terminology associated with pomegranate seeds, specifically addressing the persistent confusion in the scientific literature that incorrectly uses the term “aril” to refer to the pomegranate seed. We also highlight the techniques employed for obtaining images, characterized by their efficiency and accessibility. Macrophotography emerges as a more straightforward and cost-effective alternative compared to more complex methods, such as microscopy, which pose the risk of damaging the seed’s structure due to its high water content. With this work, our aim is to encourage a critical review of the terminology entrenched in the scientific literature, while simultaneously seeking to establish a clear standard for precise communication in the field of pomegranate research.

## 2. Materials and Methods

To empirically affirm the observations documented by Melgarejo et al. [7] and supported by historical botanical references, pomegranate fruits (*Punica granatum* L.) of the Kingdom variety were procured at three distinct developmental and ripening stages. The Kingdom variety was selected for its economic significance in the market and the distinctive coloration of its mature seeds, providing an unequivocal visual perception of their optimal state for consumption.

Thus, the Kingdom pomegranate, known for its exceptional productivity and semi-acidic nature, exhibits superior color and conformation compared to the Wonderful variety, despite sharing a similar harvest window. This selection was based on the variety’s economic significance and its alignment with consumer preferences, given its reputation for producing large fruits with vivid coloration [6].

**Figure 2:**
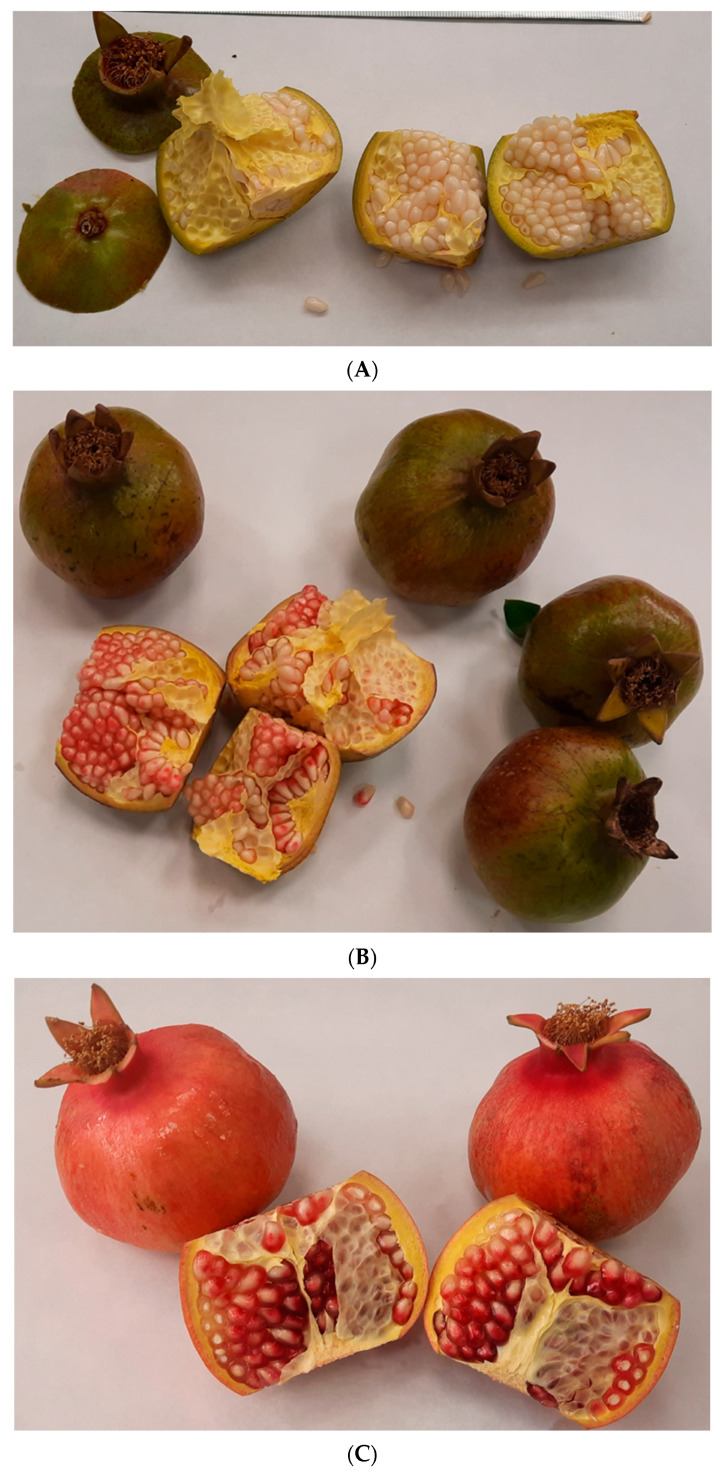
Images of the three stages of development and ripening of pomegranates studied in this work. (**A**) E1: initial development stage (June) with a fruit diameter between 2–4 cm; (**B**) E2: intermediate ripening stage (late July) with a fruit diameter between 4–8 cm; (**C**) E3: commercial ripening stage (early October) with a diameter exceeding 10 cm.

The specific harvest moments were as follows: (i) E1: the inception of fruit development, typified by the month of June, a phenological phase often marked by tree thinning, wherein the fruits remain small and predominantly green; (ii) E2: the intermediate phase of ripening, where the fruits commence their transition towards the characteristic red hue and undergo size augmentation, typically unfolding in late July; (iii) E3: the fully ripe stage, coinciding with the ultimate commercial ripening phase of fruits ready for market (early October). In E3, the fruits attain the requisite size and exhibit an intensely vibrant red coloration, as depicted in Figure 2.

Following the minimum sample size criteria, 25 fruits were manually collected from three different trees in a commercial plot for each ripening stage. Collection took place in Ojós (Murcia, Spain), considering all levels and orientations of the trees to ensure sample representativeness. At the time of collection, the trees exhibited appropriate phytosanitary conditions, and the fruits showed no damage to the pomegranate peel.

The fruits were immediately transported to the laboratory, and their evaluation occurred on the collection day. To visualize the pomegranate seeds, vertical and horizontal tangential incisions were made on the whole fruits using a sterile carbon steel surgical blade with a single cutting edge (Swann Morton, Sheffield, UK). Cuts were made using constant pressure to prevent or minimize seed damage due to water loss.

Thus, all fruits were divided into sections that allowed visualization and photographic recording of the seeds, with an emphasis on differentiating their various parts (testa, tegmen (In botany, the term “tegmen” refers to the outer covering of a seed. This protective layer can be a structure that envelops the seed, shielding it during its development and storage. The tegmen is often composed of tissues that can be either thick or thin, and its main function is to safeguard the integrity of the seed. The presence and specific characteristics of the tegmen can vary among different plant species.), and embryo with cotyledons). Evaluation was conducted in the laboratory using a Samsung WB100 camera with a 22.3 mm f/3.1–5.9 zoom lens mounted on a two-section telescopic table stand TL253A (Neewer, Shenzhen, China). For all fruit samples, more than 40 photographs were taken to confirm the observed results.

## 3. Results and Discussion

The study’s findings robustly confirm that pomegranate seeds constitute the integral grain of the edible portion of the fruit. These seeds are classified as exarilate, aligning with Melgarejo et al.’s insights [7].

A comprehensive analysis of the various stages of development and ripening highlights the intricate structure of the seed. In the initial developmental phase, prism-shaped seeds populate the fruit’s interior, each enveloped by a fleshy covering known as the testa. As the fruit ripens, part of the testa sclerotizes, acquiring a woody consistency, giving the seed its characteristic hardness. At the core of the seed, the embryo with neatly rolled cotyledons is found.

**Figure 3:**
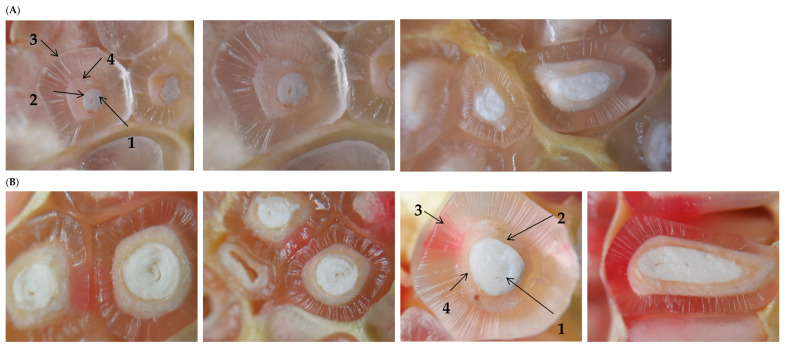

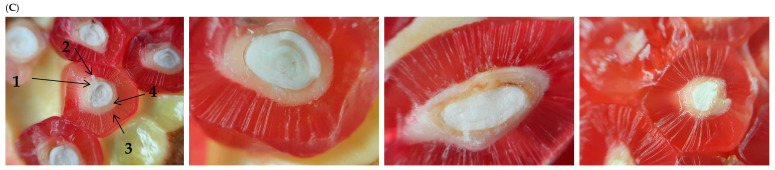
Images of the development of Kingdom pomegranate seeds during different fruit ripening stages: (**A**) The initial ripening stage (June); (**B**) Intermediate ripening stage (late July); (**C**) Commercial ripening stage (early October).(1) The embryo with cotyledons rolled upon themselves; (2) The inner membrane or tegmen; (3) The outer pulpy membrane or sarcotesta; (4) Mesotesta and endotesta.

Figure 3 serves as a visual testament to the remarkable transformations that unfold throughout the ripening process of these fruits. Within this illustration, the pomegranate seeds are distinctly portrayed as reproductive structures comprising an outer fleshy testa referred to as the sarcotesta, characterized by a single layer of translucent, columnar cells. Inward from the sarcotesta, the mesotesta (In botany, “mesotesta” refers to a specific layer in the structure of a seed. The mesotesta is part of the outer layers of the testa, which is the protective covering of a seed. The mesotesta is situated between the outer layer (exotesta) and the inner layer (endotesta) of the seed’s testa. Each of these layers may have distinct characteristics in terms of thickness, texture, and cellular composition. The presence and specific properties of the mesotesta can vary among different plant species), defined by its hard and thick characteristics, becomes the central feature. The mesotesta’s cell walls undergo a progressive thickening as the fruit matures, and these sclerotized (It refers to the process in which the cells of plant tissues develop thicker and hardened cell walls, usually composed primarily of lignin. This process contributes to the rigidity and strength of plant tissues) cells significantly contribute to the seed’s firmness when being chewed. Further inward, the tegmen takes its place, comprised of two membranous epidermal layers tinted in a yellowish-brown hue. Lastly, the seed is anchored by the embryo, with the cotyledons carefully arranged on top of one another.

The results from this investigation offer crucial insights into dynamic changes during the seed’s development and ripening. Notably, testa cells experience a remarkable increase in size and volume, culminating in the fleshy or pulpy testa observed in fully ripe pomegranates. This unique structure functions as a repository where water and an array of bioactive compounds accumulate. Additionally, the study highlights the fascinating observation of the color transition in this pomegranate variety, marking it as a noteworthy milestone in the fruit’s maturation journey.

## 4. Conclusions

In conclusion, this comprehensive study addresses common misconceptions and corrects interpretations of fundamental botanical concepts prevalent in numerous articles. It is crucial to underscore the importance of precise botanical terminology in scientific communication, as it significantly influences the understanding and dissemination of research findings. Specifically, we recommend discontinuing the use of the term “aril” to refer to the pomegranate’s seed or to describe its testa, as it is unequivocally an exarilate fruit. Instead, it is essential to acknowledge that the edible component of the pomegranate comprises the entire seed, including the testa, tegmen, and the embryo with its cotyledons.

Furthermore, the term “seed” should not be casually applied to the internal seed part of the pomegranate fruit. These findings substantiate a precise understanding of the pomegranate fruit’s intricate structure and composition, establishing a robust foundation for future research endeavors and opening up possibilities for applications across various scientific, technical, and commercial domains.

The potential implications of this new understanding for future research in botany, agriculture, nutrition, and phytotherapy are extensive. For instance, these findings may influence breeding programs, nutritional analysis, or the extraction of bioactive compounds from pomegranate seeds. Subsequent research could explore variations in seed structure or composition among different pomegranate varieties and investigate the genetic basis for these anatomical features.

Considering the broader impact of these findings on the field of plant science and related industries, such as horticulture and pharmacology, the significance of correcting these misconceptions is emphasized. This advancement in our understanding of the pomegranate and its potential utilization is pivotal.

So, this study not only corrects misconceptions but lays the groundwork for future exploration and application. It is a call to action for researchers, practitioners, and industries to adopt precise botanical terminology, fostering a more accurate and impactful discourse in the scientific community. The clarity brought by our findings contributes substantially to the field, promising advancements in multiple domains. This research is not merely a correction; it is a significant stride towards unlocking the full potential of the remarkable pomegranate.

## Data Availability

Data are contained within the article.

## References

[1] Melgarejo P., Salazar D.M. (2003). Tratado de Fruticultura para Zonas Áridas y Semiáridas (Vol. II).

[2] Lorente-Mento J.M., Guillén F., Martínez-Romero D., Carrión-Antoli A., Valero D., Serrano M. (2023). γ-Aminobutyric Acid Treatments of Pomegranate Trees Increase Crop Yield and Fruit Quality at Harvest. Sci. Hortic..

[3] Melgarejo P., Núñez-Gómez D., Martínez-Nicolás J.J., Giordani E., Tozzi F., Legua P. (2021). Fatty Acids Compositional Variations between the Edible and Non-Edible Fruit Part of Seven Pomegranate Varieties. Food Chem. Mol. Sci..

[4] Ministerio de Agricultura, Pesca y Alimentación—MAPA (2022). Superficies y Producciones Anuales de Cultivos.

[5] Stiletto A., Trestini S. (2021). Factors behind Consumers’ Choices for Healthy Fruits: A Review of Pomegranate and Its Food Derivatives. Agric. Econ..

[6] Tozzi F., Legua P., Martínez-Nicolás J.J., Núñez-Gómez D., Giordani E., Melgarejo P. (2020). Morphological and Nutraceutical Characterization of Six Pomegranate Cultivars of Global Commercial Interest. Sci. Hortic..

[7] Melgarejo P., Núñez-Gómez D., Legua P., Martínez-Nicolás J.J., Almansa M.S. (2020). Pomegranate (Punica granatum L.) a Dry Pericarp Fruit with Fleshy Seeds. Trends Food Sci. Technol..

[8] Molla S.M.H., Rastegar S., Omran V.G., Khademi O. (2022). Ameliorative Effect of Melatonin against Storage Chilling Injury in Pomegranate Husk and Arils through Promoting the Antioxidant System. Sci. Hortic..

[9] Cheng J., Li J., Xiong R.-G., Wu S.-X., Huang S.-Y., Zhou D.-D., Saimaiti A., Shang A., Feng Y., Gan R.-Y. (2023). Bioactive Compounds and Health Benefits of Pomegranate: An Updated Narrative Review. Food Biosci..

[10] Melgarejo-Sánchez P., Núñez-Gómez D., Martínez-Nicolás J.J., Hernández F., Legua P., Melgarejo P. (2021). Pomegranate Variety and Pomegranate Plant Part, Relevance from Bioactive Point of View: A Review. Bioresour. Bioprocess..

[11] Ashtari M., Khademi O., Soufbaf M., Afsharmanesh H., Askari Sarcheshmeh M.A. (2019). Effect of Gamma Irradiation on Antioxidants, Microbiological Properties and Shelf Life of Pomegranate Arils Cv. ‘Malas Saveh’. Sci. Hortic..

[12] Velotto S., Palmeri R., Alfeo V., Gugino I.M., Fallico B., Spagna G., Todaro A. (2023). The Effect of Different Technologies in Pomegranate Jam Preparation on the Phenolic Compounds, Vitamin C and Antioxidant Activity. Food Biosci..

[13] Martínez-Nicolás J.J., Hernández F., Núñez-Gómez D., García-Sánchez F., Martínez-Font R., Legua P., Melgarejo P. (2023). Metabolomic Approach to Study the ‘Purple Queen’ Pomegranate Cultivar Response to Alternative Culture Media and Phenological Stages. Foods.

[14] Komali N.D., Gaikwad P.S., Yadav B.K. (2022). Fabrication of Cellulose Acetate Membrane for Monitoring Freshness of Minimally Processed Pomegranate (Punica granatum) Arils. Food Biosci..

[15] Umdale S.D., Patil P.D., Malik S.K., Latha M., Rao S.R., Yadav S.R., Gaikwad N.B., Bhat K.V. (2017). Seed Coat Sculpture of Subgenus Ceratotropis (Piper) Verdc., Genus Vigna Savi in India and Its Taxonomic Implications. Bot. Lett..

